# Anthropogenic modifications: impacts and conservation strategies

**DOI:** 10.1038/s41598-023-38940-x

**Published:** 2023-07-25

**Authors:** Andrea Belgrano, Federica Cucchiella, Dong Jiang, Marianna Rotilio

**Affiliations:** 1 Department of Aquatic Resources, Institute of Marine Research, Swedish University of Agricultural Sciences, Uppsala, Sweden; 2 Swedish Institute for the Marine Environment (SIME), University of Gothenburg, 405 30 Gothenburg, Sweden; 3 University of L’Aquila, L’Aquila, Italy; 4 Institute of Geographic Sciences and Natural Resources Research, CAS, Beijing, China

**Keywords:** Environmental sciences, Environmental social sciences

Humans have become one of the primary forces shaping our planet, altering climatic, geological and biological systems at an unprecedented pace. A whole new geological period has been proposed, the Anthropocene, which highlights just how much of a priority the reduction and management of anthropogenic pressures are becoming globally. Within the topic, this collection brings together studies on the analysis of impacts modifying existing ecosystems, as well as mitigation and conservation strategies. It allows for the development of reflections on potential problems and good practices already identified.

The Anthropocene is a timely topic on which researchers are urgently debating, defining it as a complex, heterogeneous and currently ongoing event^1^, which may be assigned the rank of series/epoch^2^. In reality, the Anthropocene has yet to be formally defined, with the application of standard protocols over a long period of time^2^, but it appears evident how the actions and impacts of human beings, as well as the built environment, has an important role in this ongoing transformation^3,4^.

This Collection gathered contributions that deal with the theme mainly from two different points of view. On the one hand, the impacts and alterations of natural systems generated by human action are analysed. On the other hand, mitigation and conservation strategies have been researched that can also be replicated in other contexts.

With reference to the first of the two approaches, the contribution of Rafeeque et al. examines the role of anthropo-geomorphic interventions that have caused the degradation of the territory of Munroe Island (India)^5^. A multidisciplinary method is employed which exploits the contribution of multi-dated, multiresolution satellite products and published maps over a period of approximately sixty years. The study demonstrates the perturbation that has occurred in the region over the years which has led to the disappearance of 14% of the island’s surface, as well as significant degradation, and identifies the direct cause in human-induced hydrogeomorphic interventions. Forti et al. analyse the erosive processes that threaten the preservation of archaeological tell sites in arid regions due to ongoing climate change and land use for grazing purposes. They apply the revised universal soil loss equation model for soil loess using UAV imagery and geoarchaeological investigation^6^. Tjiputra et al. also used satellite data to analyse the variation of oxygen, acidity (pH), temperature, and salinity from the surface up to a depth of 2000 m in the global ocean^7^. In their study they demonstrate how anthropogenic changes emerge earlier in the internal ocean than at the surface, due to the lower background variability at depth. Thus, even in mitigation scenarios, anthropogenic signals detected within the ocean will emerge in the coming decades. The researchers highlight the need to establish systems for monitoring the interior to understand the impact of marine ecosystems and biogeochemistry. With an equal focus on the marine system, particularly the coastal one, Alldred et al. used macroalgae as a bioindicator of the anthropogenic nitrogen load on the coasts of the United Kingdom^8^. The authors compared two island systems, Jersey (Channel Islands) and St Mary's (Isles of Scilly), to assess how different sewage infrastructure affects nitrogen loading and demonstrated that upgrading sewer systems in the islands is necessary to reduce environmental problems. Two studies focused their attention instead on the soil^9,10^. Jungkunst et al. highlight how the relationship between human actions and the presence of organic carbon (SOC) in soil in the Anthropocene is vague, mainly due to the lack of comparison between pristine sites and sites where human activities are present^9^. Thanks to their study, conducted on an intact site in the high Andes, they provide evidence that thousands of years of pastoralism have increased the persistence of carbon in the soil. de Silva et al. found that in Asia, since 1700, more than 64% of suitable elephant habitat has been lost in conjunction with land use practices for agricultural purposes^10^. Finally Sor et al. show how in the Lower Mekong Basin, dams built to produce renewable hydropower reduce fish biodiversity^11^. They therefore recommend using existing dams rather than building new ones, as well as diversification in the development and use of renewable energy sources.

In a second group of articles in the Collection, the Anthropocene was studied with the aim of identifying mitigation and conservation strategies that can also be replicated in other contexts: soil and forests are the most sensitive ecosystems. Mishra et al. analyse the effectiveness of water and soil retention structures called crescents, built to restore degraded land in Niger. The pre and post-intervention analysis showed that crescents effectively adapt traditional land management systems to increase agricultural production in arid ecosystems and are easily replicable systems in other contexts^12^. Brandolini et al. illustrate a multidisciplinary approach to demonstrate how pre-industrial agricultural elements can mitigate soil erosion risk in response to current environmental conditions^13^, while Ferrer Velasco et al. analyse the opinions of forest stakeholders on the policies implemented to fight deforestation^14^. Finally, Österblom et al. focus on the biosphere, highlighting the need to implement sustainable changes in company policies relating to fish products^15^. Thanks to the support of scientists, it was possible to implement collaborative practices to improve best approaches in ocean management. The importance of this contribution lies mainly in demonstrating that cultural evolution is the basis of any action aimed at mitigating impacts and protecting the planet^15^.

In conclusion, this collection includes studies on the topic of the Anthropocene, which has been proposed as a new geological era but is still being formally defined. The topic is mainly treated from two points of view, that of ecosystem impacts and transformations, and on the other hand, that of the definition of mitigation and conservation strategies for existing balances. The main areas addressed are those concerning the transformation and degradation of soil properties, landscape conservation, the marine ecosystem as a whole and the coastline, mainly in relation to human action. It is evident how the collection acts as a pathfinder for very important topics but only delves into some of the main areas of existing ecosystems. It may therefore give rise to new lines of research in relation to the other areas of the ecosystems addressed here. Finally, it allows for the development of reflections on potential problems and good practices already identified.
